# Influence of Multi-Holes on Fatigue Behaviors of Cast Magnesium Alloys Based on In-Situ Scanning Electron Microscope Technology

**DOI:** 10.3390/ma11091700

**Published:** 2018-09-13

**Authors:** Xi-Shu Wang, Chang-Hao Tan, Juan Ma, Xiao-Dong Zhu, Qing-Yuan Wang

**Affiliations:** 1 School of Mechanical Engineering, Chengdu University, Chengdu 610106, China; jma10s@alum.imr.ac.cn (J.M.); xiaodangjia21@126.com (X.-D.Z.); 2 Department of Engineering Mechanics, Tsinghua University, Beijing 100084, China; tch17@mails.tsinghua.edu.cn; 3 Department of Civil Engineering & Mechanics, Sichuan University, Chengdu 610065, China

**Keywords:** cast magnesium alloy, hole and notch, fatigue crack, fatigue life, in-situ SEM technology

## Abstract

The low cycle fatigue tests on the crack initiation and propagation of cast magnesium alloys with two small holes were carried out by using in-situ scanning electron microscope (SEM) observation technology. The fatigue crack propagation behaviors and fatigue life, which are affected by two small artificial through holes, including the distances between two holes and their locations, were discussed in detail based on the experimental results and the finite element analysis (FEA). The results indicated that the fatigue multi-cracks occurred chiefly at the edges of two holes and the main crack propagation was along the weak dendrite boundary with the plastic deformation vestiges on the surface of α-Mg phase of cast AM50 and AM60B alloys. The fatigue cracking characteristics of cast AZ91 alloy depended mainly on the brittle properties of β-Mg_17_Al_12_ phase, in which the multi-cracks occurred still at the edges of two holes and boundaries of β-Mg_17_Al_12_ phase. The fatigue crack initiation position of cast magnesium alloys depends strongly on the radius of curvature of through hole or stress concentration factor at the closed edges of two through holes. In addition, the fatigue multi-cracks were amalgamated for the samples with titled 45° of two small holes of cast Mg-Al alloys when the hole distance is less than 4D (D is the diameter of the small hole).

## 1. Introduction

As one of the light metals, the magnesium (Mg) alloys, such as AM50, AM60B, AZ31 and AZ91, have such a high strength-to-weight ratio that they are widely suitable for ultimate weight reduction purposes in automotive and aircraft components as well as other applications [1,2,3]. As most of magnesium alloys are from the foundry and rolling processing technologies, the microstructural defects and other notches of material or structure will inevitably exist in the material interior and surface. In addition, these alloys have been widely used for the plates with small holes in the automotive and aircraft industry in order to be riveted [4,5]. Therefore, it has stimulated the substantial interest in understanding their structural integrity and service safety of components by using the magnesium alloys including the microstructural characteristics and mechanical properties in the last decade [6,7,8], enhancing strength and thermal crack resistance of cast magnesium alloys [9,10,11], the improving elongation and low-cycle fatigue (LCF) behaviors of magnesium alloys by means of grain refinement and hot-rolled treatment methods in which the deformation mechanism of AZ31 or AZ91 alloy depends on the grain size [12] and pre-compression deformation process but these magnesium alloys are most limited in the deformed magnesium aluminum alloys, especially AZ31 alloy [13,14,15]. When the average grain (α-phase) size is about 1–2 μm, the coupling mechanism of rolling and sliding deformation of these grains occurred under the applied tensile loads so that the elongation of AZ31 alloy has been more incremental than that for the only sliding deformation mechanism of AZ31 alloy with over than 50 μm of grain size [12]. Therefore, with enhancing or improving of strength and elongation of cast magnesium alloys, the topics related to fatigue behaviors of cast or semi-solid magnesium alloys, including the fatigue cracking mechanism, fatigue life model, empirical formula of fatigue crack growth rate, reliability evaluation of fatigue data and so forth, have been widely explored. For example, Gall et al. revealed that the environment induced the fatigue crack propagation based on the cross-section observation of cast magnesium alloy [16], Wang et al. reported that the in-situ SEM measured the results which the fatigue cracking mechanism and the fatigue crack evolution process of cast AM50 alloy including the effect of high temperature on the fatigue crack growth rate [17,18,19]. The results showed clearly that the fatigue crack initiation and propagation occurred mainly in the β-Mg_17_Al_12_ and along the interface propagation between the α-phase and β-phase through the in-situ SEM observation. That is to say, the LCF fracture of cast Mg-Al alloys is an intergranular fracture at room temperature [17,18,19] but is a hybrid of intergranular and transgranular fracture at the elevated temperature [18]. Li and Peng et al. described also the relationship between the tensile strength, surface hardness, fatigue life and characteristic of cast AZ91D [20]. Horstemeyer’s group investigated the high cycle fatigue problems of cast AM50 and AZ91 E-T4 magnesium alloys and simulated the fatigue crack growth in a die-cast AM50 magnesium alloy based on the micro analysis method [21,22]. Later the substantial experimental observations about cast Mg-Al alloys have shown how the dendrite cell size, pores, shape of secondary phase particles, persistent slip bands and twinning in the dendrite cells influence on the fatigue durability and crack growth of dendritic Mg-Al alloys under different testing conditions [17,18,19,21], especially at the high temperatures [18]. Despite the importance of numerical simulations in the development of robust and cost-effective manufacturing and design methodologies, efforts aimed at examining. These microstructures play role in influences of the fatigue crack initiation and propagation. Therefore, Mayer et al. [23] and other material scientists [24,25] studied the effects of processing technics and humidity on the fatigue behaviors or limit of cast magnesium alloys [26,27], especially Murugan et al. [28] and Mohd et al. partly [29] discussed the statistical characteristics of the high cycle fatigue data of cast magnesium alloys according to the fatigue life and microstructural pore size of cast magnesium alloys. In addition, the numerical simulation studies on the fatigue micro-crack initiation or propagation path of metal including simulation method or technology (such as XFEM) were also developed in recent [30,31,32]. These simulation results assist deeply to understand the induced fatigue crack initiation and propagation reasons of materials. 

The previous research considered seldom the multi-holes in submillimeter scale and their influence on the fatigue crack initiation and propagation path of cast Mg-Al alloys. Moreover, small through holes are very common in cast Mg-Al alloys and they are regarded as major impediment in obtaining good mechanical performance from cast components for the actual design applications. Wang et al. reported roughly the fatigue crack initiation position and fatigue fracture results of cast AM60B and AZ91 alloys with two holes in the previous conference article [33]. However, what to cause the reasons of fatigue crack initiation and how to influence on the fatigue crack propagation path of cast AM60B and AZ91 alloys were not clearly and perfectly described in the previous literature [33]. In this work, the reasons of two holes influence on both the fatigue crack initiation and propagation behaviors of cast AM60B and AZ91 alloys were explained in detail by using in-situ SEM technology and numerical simulation method. Far more important is that the quantitative relationship between fatigue life and fatigue crack growth rate of cast Mg-Al alloys with two holes were not investigated. Therefore, the fundamental purpose of current work aims to explain in detail how to do the two holes (including single notch) effect on the fatigue behavior of cast AM50, AM60B and AZ91 alloys based on enough proofs in both fatigue crack developmental processes and theoretical analysis. For instances, for an AM50, AM60B, AZ31 and AZ91 alloys, the influences of multi-holes in cast Mg-Al alloy plate on the fatigue crack initiation mechanism and propagation path including the critical distance of multi-holes were clearly illustrated. In addition, the influences of two holes in different locations on the fatigue small crack initiation and propagation characteristics of cast Mg-Al alloys were discussed and the difference of fatigue life both cast AM60B and AZ91 alloys under the same condition is an equal proportion of the difference of fatigue crack growth rate both cast AM60B and AZ91 alloys. 

## 2. Experimental and Material

**Figure 1:**
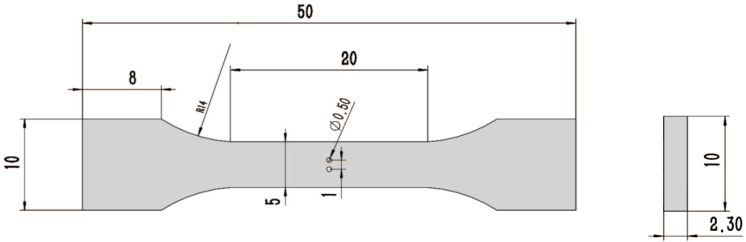
Schematic illustration and size of the specimen; all dimensions’ unit: mm.

**Figure 2:**
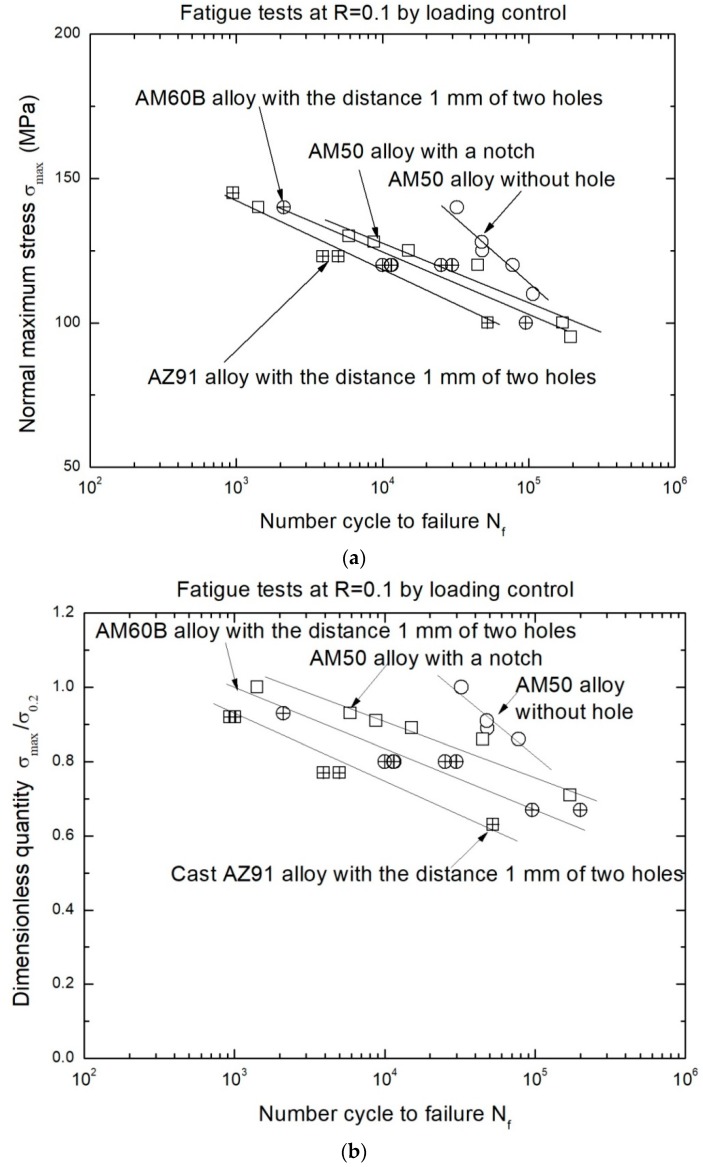
S-N curves of cast Mg-Al alloys under different testing conditions. (**a**) S-N curves of cast Mg-Al alloys with the σ_max_ parameter; (**b**) S-N curves of cast Mg-Al alloys with the λ (σ_max_/σ_0.2_) parameter.

The plate specimens for the cast AM50, AM60B and AZ91 alloys used in this study were supplied by Norsk Hydro Inc. (Oslo, Norway). Their chemical compositions and main mechanical properties are listed in Table 1. The flat cast of the Mg-Al alloy was processed into the experimental specimens with a 20 mm gage length and about 4.5–5.0 mm (width) by 2.3 mm (thickness) gage cross section by using a wire-electrode cutting method. Every specimen has two through holes in the middle region and the diameter of every through hole is about 0.5 mm, in which the spatial distance (λ) between two through holes is 1.0 mm (2D), 1.5 mm (3D), 2.0 mm (4D) and 3.0 mm (6D), respectively. The tilted angle, α, is defined as the angle between the line connecting the center of two holes and the applied loading direction. In this work, two values, that is, α = 45° and α = 90°, were considered for the tilted angle, as shown in Figure 1. The two through holes were fabricated by the mechanical drilling method from Westmoreland Mech. Testing & Research, Inc., Youngstown, PA, USA in order to avoid the heat affecting. Therefore, the edge of a through hole has still some micro notches to be caused by the slight vibration in the drilling process. Therefore, the fatigue data of cast AM50 alloy with and without a single notch were compared with that of other cast Mg-Al alloys under the different conditions as shown in Figure 2. To investigate the effect of multi-holes on fatigue behavior of cast AM60B and AZ91 alloys, the in-situ SEM (by Shimadzu, Kyoto, Japan) observation technology must be used in this work. The detail results were shown in the next section of analysis or discussion. 

The observation faces of all samples were carefully etched in the NH_4_Cl saturated aqueous solution after polished by abrasive paper (2000 grit) to achieve a surface roughness, *R*a, of about 0.8–1.0 µm. All fatigue crack initiation and propagation tests were carried out in the vacuum chamber of the SEM with specially designed servo-hydraulic testing system by Shimadzu, Kyoto, Japan. This machine provides a pulsating (sine wave) load at 10 Hz with a ±1 kN maximum capacity and a displacement range of ±25 mm. The signal of the SEM was directly transferred to a computer via a direct memory access type A/D converter, making it possible to sample 960 × 1280 frames of SEM images successively. The SEM was operated at an accelerating voltage of 15 kV in order to obtain clear images. As the projection length of the fatigue micro-crack is different under opening and close states, it was measured directly with the calibrated scale of SEM for only the opening state in this work [19]. The whole process of the fatigue crack initiation and propagation was monitored and recorded in-situ at a frequency of 0.01 Hz. The stress ratio *R* is 0.1 and the fatigue tests are controlled by loading [17,18,19].

## 3. Fatigue Experimental Results

The fatigue data of the cast magnesium alloys (AM50, AM60B and AZ91) under different conditions is shown in Figure 2, where the cases for AM60B and AZ91 were chosen as one of the typical results with two through holes for spacing 1.0 mm, 45° tilted to the applied loading direction. And the fatigue data of AM50 alloy in the S-N curves were in order to compare with the difference of two types samples with a single notch and without hole or single notch, in which its radius of curvature is about 0.5 mm and the depth of notch is about 0.6 mm to be equivalent to the diameter of a small hole by using mechanical machining method. Figure 2a indicates that the fatigue performance of the AZ91 alloy is the lowest among three cast magnesium alloys but the S-N curves for the three cases (AM50, AM60B and AZ91) exhibit similar changing trend with roughly the same slope for different artificial defects including notch and holes. The differences of the fatigue life/performance in these S-N curves depend mainly on both the yield stresses of materials and artificial defect types. It reflects the fact that the raising of the fatigue life is with the increase of elongation of cast magnesium alloys. It also hints that the LCF damage behavior of cast Mg-Al alloys may be dominated by the deformation capability but not the static tensile strength of cast Mg-Al alloy as shown in Table 1. Therefore, one of potential ways to enhance the fatigue performance is to improve the plastic deformation capability of cast Mg-Al alloys. For example, by changing the β-phase (Mg_17_Al_12_) size or sharp or the refining the α-phase grain the plastic property of cast Mg-Al alloys can be improved [12,13,14,15]. In addition, the effect of deflects (such as single notch and multi-holes) on the fatigue performance of cast Mg-Al alloys cannot be ignored, as shown in Figure 2a. In order to reduce the influence of the yield stresses or elongation rates on the fatigue performance of cast Mg-Al alloys, the relationships between relative fatigue strengths (defined as λ = σ_max_/σ_0.2_) versus the number cycle to failure of three cast Mg-Al alloys were plotted for the alloys. It can be found that the fatigue performance of cast AM60B alloy is superior to that of cast AZ91 alloy under the same condition. By comparing the effects of the single notch and the two holes with a distance 1.0 mm and a tilted angel of 45° on the relative fatigue performance, we see that the relative fatigue performance of cast AM50 alloy with a single notch is obviously better than that of AM60B alloy which has two small holes with the distance of 1.0 mm as shown in Figure 2b but the difference of fatigue performance both single notch and distance λ = 1.0 mm, tilted 45° of two small holes is not obviously in Figure 2a.

## 4. Analysis and Discussion

### 4.1. Effect of Tilted Angles of Multi-holes on Fatigue Behavior of Cast AM60B Alloy

**Figure 3:**
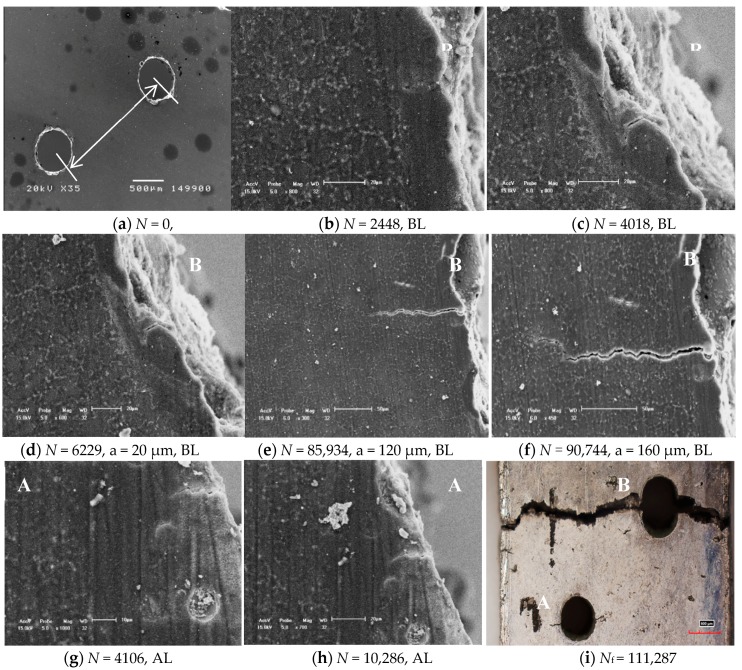
Fatigue crack initiation and propagation behaviors of cast AM60B alloy with tilted 45° and hole distance 1.5 mm (3D) at the maximum stress level of 100 MPa, *R* = 0.1, *N*_f_ = 111,287.

**Figure 4:**
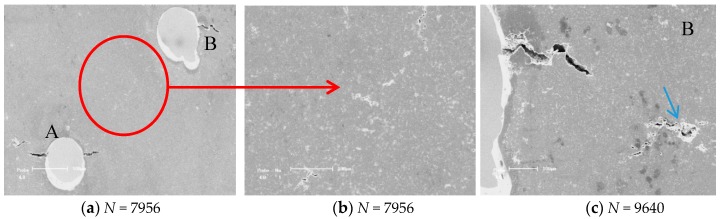
Fatigue crack initiation and propagation behavior of cast AM60B alloy with tilted 45° and hole distance 2.0 mm (4D) at the maximum stress level of 120 MPa, *R* = 0.1, *N*_f_ = 61,562. (**a**) *N* = 7956, (**b**) *N* = 7956, (**c**) *N* = 9640.

**Figure 5:**
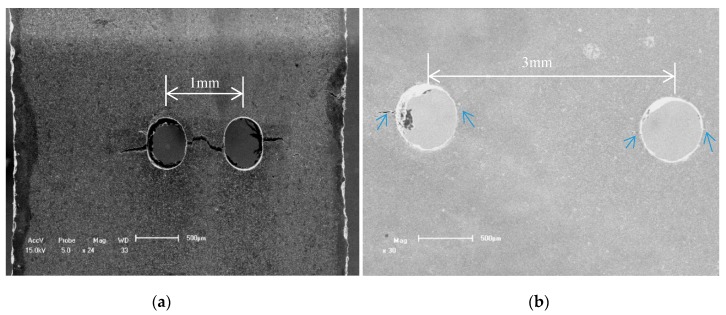
Fatigue crack initiation and propagation behavior of cast AM60B alloy with tilted 90° and different hole distances of two small holes. (**a**) *N* = 9446 (*N*_f_ = 17,433), σ_max_ = 120 MPa, 2D, *R* = 0.1. (**b**) *N* = 25,046 (*N*_f_ = 25,202), σ_max_ = 120 MPa, 6D, *R* = 0.1

In order to quantitatively to explain the effects of the distances between the two holes distance and the tilted angles on the differences of the LCF life of cast AM60B alloy, the results for a typical fatigue crack initiation and propagation characteristics of cast Mg-Al alloys are shown from Figure 3, Figure 4, Figure 5, respectively. For example, the fatigue crack initiation and propagation characteristic for cast AM60B alloy with the distance *λ* = 1.5 mm (3D) and α = 45° as a typical case is shown in Figure 3. The in-situ SEM measured results showed that the tilted angle of the two holes affects significantly the fatigue crack behaviors of cast AM60B alloy at the stress level of 100 MPa and the stress ratio *R* = 0.1. When the cyclic number is *N* = 2448, a small crack with an initiation length of about 15 μm occurred at the root of the closed notch on the left side of Hole B (denoted by BL) as shown in Figure 3a,b, while no small crack was found on both sides of Hole A after about 2500 cycles. Therefore, the fatigue crack initiation time exists in the precedence for the Hole A and Hole B, in which there are still the different burrs on the edge of a through hole caused by the slight vibration in the drilling process as shown in Figure 3a. However, after *N* = 4018 and *N* = 4106 cycles respectively at BL and at the edge of Hole AL (the left side of Hole A) seats as shown in Figure 3c–f, these fatigue crack propagation behaviors indicated that the fatigue crack propagation path at BL deviated obviously from the previous crack initiation direction. In addition, although the fatigue crack at the left side of Hole A was found after *N* = 4106 cycles or *N* = 10286 cycles, the crack growth rate at the two sides of Hole A were much lower compared to the crack lengths as shown in Figure 3g,h. At the same time, when *N* = 111,287, the fatigue crack growth lengths at the two sides of Hole A are respectively about 180 μm and 200 μm but another fatigue crack length at the left side of Hole B has extended about several millimeters until the fatigue fracture of this specimen as shown in Figure 3i. This means that the fatigue crack initiation mechanism of cast AM60B alloy is similar to that of cast AM50 alloy [16,17,18,19], both called the grain boundary crack initiation mechanism in micro scale. At the same time, the fatigue cracks at the location of AL initiated with a length of about 10 μm and propagated to about 20 μm after 4106 cycles and 10,286 cycles as shown in Figure 3g,h, respectively. At the same time, this result indicated that the two holes with the distance λ = 1.5 mm (3D) did not induce the fatigue cracks amalgamation but the crack initiation behavior is influenced by the stress concentration at the root of the corresponding notch. With increasing of the cyclic number, the fatigue crack at AL hole stopped the propagation as shown in Figure 3h, while the fatigue crack at BL was found from Figure 3d–f to extend rapidly. After 111,287 cycles, the specimen broke and the final fatigue fracture pattern is shown in Figure 3i. This case indicated that the effect of two holes, which have a tilted angle of 45° and a distance of 3 times their radius, on the fatigue crack propagation path can be ignored. For example, the fatigue crack initiation and propagation vestige of cast AM60B alloy was not found in the connection area of two holes. The fatigue fracture of sample was contributed by the fatigue crack propagation of Hole B, especially the fracture of specimen was caused by the fatigue crack propagation at the left side of Hole B as shown in Figure 3i. However, another case of fatigue crack initiation and propagation images under a higher stress level (120 MPa) indicated that effect of 45° tilted and spacing 4D of two holes (Hole A and Hole B) cannot be ignored as shown in Figure 4a. When the cycle is after 7956 cycles, at the maximum stress level of 120 MPa, *R* = 0.1, the LCF crack propagation behavior can be simply described by the early stage of fatigue crack propagation occurred still at every notch root of two small holes as shown at Hole A and Hole B in Figure 4a. The LCF crack at Hole A occurred in two sides of hole and LCF crack at Hole B occurred in the starboard of hole. And these fatigue cracks growth directions are basically along the projection direction of applied loading. But there are much more fatigue small cracks in the linked area of 45° tilted to the applied loading direction both two small holes as shown in Figure 4b, in which the propagation directions of these fatigue small cracks appeared disorganized. With increasing of cycle (*N* = 7956 cycles), the fatigue multi-cracks occurred at Hole B in the right side of sample but these cracks were not linked as shown in Figure 4c. At the same time, the fatigue multi-cracks in the coalescence region have been linked so that the fatigue fracture pattern is observably different from the fracture result as shown in Figure 3i. The fatigue fracture between two holes was subjected by the shear stress in which it was induced by the plastic deformation when the relative strength ratios (λ = σ_max_/σ_0.2_) is over than 0.800. It hints that the fatigue cracking mechanism of cast AM60B alloy under low cycle and high cycle may be different. When λ is less than 0.7, the interface fatigue crack initiation might be occupied a dominant position and the effect of 45° tilted and holes distance over than 3D on the fatigue crack initiation and propagation path can be ignored. The fatigue crack initiation was still dominated by the stress concentration such as the radius of curvature of a notch so that the coalescence behavior of the fatigue cracks propagation did not occur at the influence area of two small holes when the distance of two holes is 3D. However, when λ is over than 0.8, the fatigue crack propagation was dominated by the plastic deformation. Even if it is without the stress concentration region, the fatigue cracks occur also in the holes coalescence as shown in Figure 4b, in which influence reason refers the next section of finite element analysis.

Another typical case of the fatigue cracking characteristics of cast AM60B alloy is showed in Figure 5, in which there are the changes of tilted angle and different distances of two small holes compared with above mentioned case of the fatigue crack characteristic of cast AM60B alloy. These results indicated that the effect of 90° tilted to the loading direction and distance 2D or 6D between two small holes on the fatigue crack initiation and propagation behavior can be defined as the same of fatigue damage model (Mode-I). After 9446 cycles, the fatigue crack initiation and propagation has been occurred at every root of notch and the fatigue cracks have been linked between two holes as shown in Figure 5a. However, with the increasing of distance between two small holes, the increasing of fatigue life is obvious as shown in Figure 5b, in which multi-cracks occurred respectively at the sides of two holes when *N* = 25,046 cycles. After 25,046 cycles, the LCF cracks between two holes were not linked but the fatigue fracture occurred fleetly between two holes when the cycle increased to 25,202 cycles. It is obvious that the smaller distance (D) between two holes is, the short of LCF life is. In addition, the fatigue crack initiation and propagation behavior depends strongly on the distance of two small holes, 45° or 90° tilted to the applied loading direction and the applied stress amplitude because of the stress concentration degree or fatigue damage model difference.

### 4.2. Effect of Tilted Angles of Multi-holes on Fatigue Behavior of Cast AZ91 Alloy

**Figure 6:**
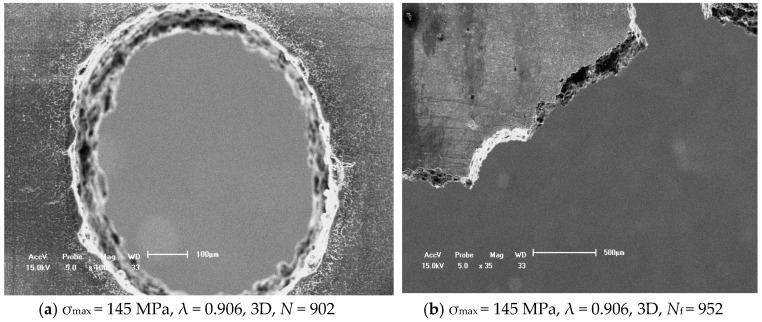
Effect of 45° tilted and distance 1.5 mm of two through holes on the fatigue crack propagation behavior of cast AZ91 alloy.

Figure 6 showed another typical case of fatigue initiation and propagation characteristics of cast AZ91 alloy under the σ_max_ = 145 MPa, *R* = 0.1, 1.5 mm (3D), in which the result indicated that fatigue crack initiation occurred still at the edge of each small hole when the cycle is 902 cycles as shown in Figure 6a. The fatigue crack propagation path in the linked region of two small holes was fleetly amalgamated as shown in Figure 6b so that the fatigue life of cast AZ91 alloy with two holes was rapidly decreased. An important reason is due the elongation of cast AZ91 alloy is about 3.5% and less than of cast AM60B alloy as shown in Table 1. As above mention the fatigue damage of cast Mg-Al alloy, the fatigue damage behavior of cast AZ91 alloy when λ is over than 0.700 is similar to that of cast AM60B alloy in which the fatigue damage of cast AM60B or AM50 alloy is mainly dominated by the plastic deformation as the result of mismatch of deformation in the interface both α-Mg and β-Mg_17_Al_12_. It is due the plastic deformation of cast AZ91 alloy is relative smaller, the LCF crack initiation might occur in randomly but these fatigue multi-cracks in the linked region of two small holes were amalgamated. Therefore, the effect of multi-holes on the fatigue crack propagation behavior of cast AZ91 alloy cannot be ignored. In addition, the fatigue cracks to occur at the notch roots of each hole propagated preferentially between the two holes from statistical data and propagated concurrently along a projected direction as shown in Figure 6b. Compared with the results above mention, whether the fatigue crack propagated of cast AZ91 alloy with the tilted 45° of two small holes not only depends on the distance of two small holes but also on the applied maximum stress amplitude or plastic strain capability. If the greater applied stress amplitude is, the greater in the probability of the fatigue crack propagated between two small holes is.

**Figure 7:**
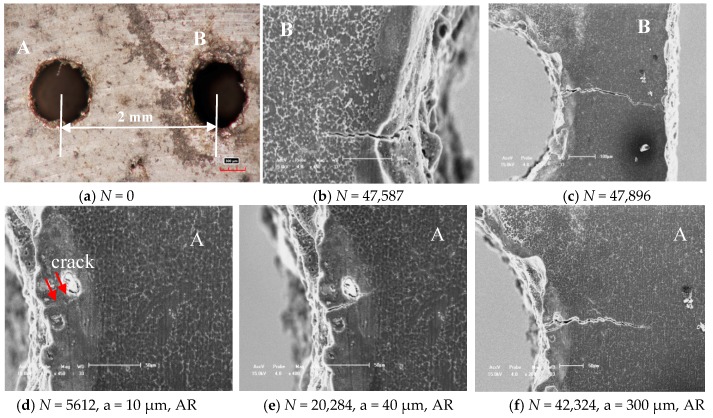
Effect of 90° titled and distance 2.0 mm of two through holes on the fatigue crack initiation and propagation of cast AZ91 alloy at the maximum stress level of 100 MPa, *R* = 0.1.

As the effect of tilted angles (α) on the fatigue crack initiation and propagation behavior of cast AZ91 alloy, it is similar to that of cast AM60B alloy when the tilted 90° and two holes distance λ = 2.0 mm (4D) as shown in Figure 6 and Figure 7, respectively. These results indicated that no matter what the distance between two small holes is, the fatigue crack initiation occurred successively and randomly at the root of holes as shown in Figure 6a and Figure 7b,c, in which the experimental conditions are after *N* = 902 cycles, at σ_max_ = 145MPa, *R* = 0.1, *N* = 47,587 cycles, 47,896 cycles, at σ_max_ = 100 MPa, *R* = 0.1, respectively. For example, the LCF cracks at left and right sides of Hole B occurred after N = 47,587 cycles, 47,896 cycles respectively. Its length is respectively about 110 μm as shown in Figure 7b and 400 μm as shown in Figure 7c. Especially, the LCF crack growth length in right side of Hole B is about four times than that in left side of Hole B as shown in Figure 7c. One of reasons is the boundary effect of sample as the LCF crack asymmetric propagation behavior in the Hole B. In addition, there are the different burr formation vestiges on the edges of Hole A and B as shown in Figure 6a and Figure 7a. These burrs are an important influence factor of the early fatigue crack initiation and propagation. Once the fatigue crack was evolved into the macro crack (such as over than 1–2 mm) length including the contribution of two through holes, the fatigue fracture of cast AZ91 alloy is easily to occur. This is due these fatigue cracks were fleetly combined in the projection direction of applied loading as shown in Figure 7c, in which *N*_f_ is 52,522. In order to further to evaluate the fatigue crack growth rate at the right side of Hole A (AR) under the above mentioned experimental conditions, the fatigue crack lengths after the different cycles were shown in Figure 7a,d–f by using in-situ SEM observation technology. Why did choose the LCF crack propagation behavior at the right side of Hole A as shown in Figure 7a? It is because of without considering the influence of the edge of sample and holes on the fatigue crack propagation in which the influence of the sample edge is greater than that of two holes when the distance of two holes is over than 2 mm (4D). Due the width of sample is about 4.5–5.0 mm in this work, the distance between Hole A or Hole B and edge of sample is less than 1.5 mm if the distance of two holes is over than 2 mm. After *N* = 5612, the LCF crack initiation length is about 10 μm as shown in Figure 7d at the root of notch for Hole A. With increasing of the cycle (*N* = 20,284, 42,324, respectively), the fatigue crack propagation length extends to about 40 μm and 300 μm as shown in Figure 7e,f, respectively. Compared with the above mention changes of cycles and crack lengths as shown in Figure 7d–f, the estimated fatigue crack growth rate of cast AZ91 alloy is about 1.38 × 10^−8^ m/cycle within 300 μm under the applied stress of 100 MPa, *R* = 0.1. And the fatigue crack growth rate of cast AM60B alloy is approximately estimated to about 0.83 × 10^−8^ m/cycle within 300 μm at the applied stress level of 100 MPa, *R* = 0.1 without also considered the effect of two small holes on the fatigue crack propagation behavior according to the results in Figure 3e,f. Therefore, the fatigue crack growth rate of cast AZ91 alloy is about slightly two times than that of cast AM60B alloy which verified that the results in S-N curves is creditability as shown in Figure 2b.

### 4.3. Validation of Fatigue Behavior by Using Finite Element Method

**Figure 8:**
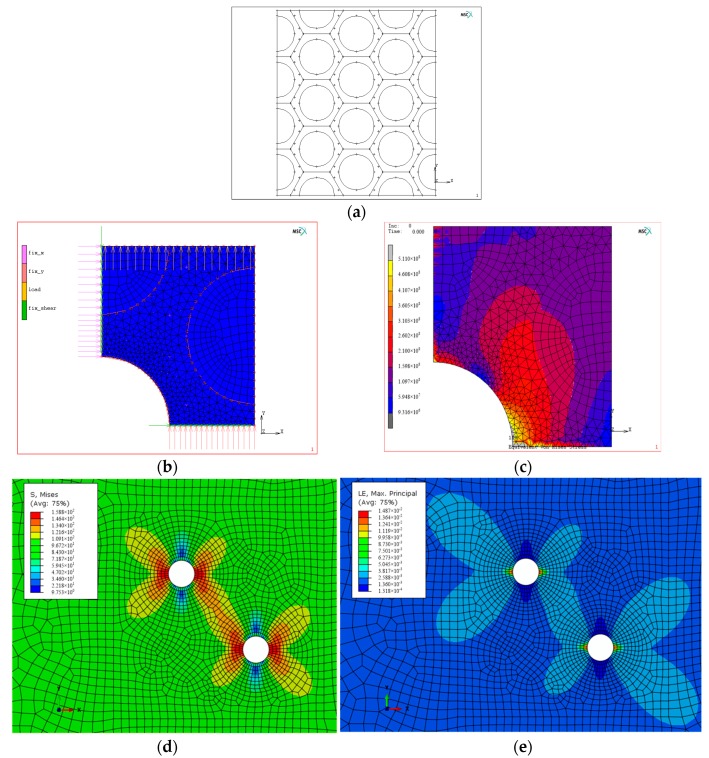
Contour plots of different stresses and plastic strain of cast AM60B alloy. (**a**) FE modeling of cellular texture; (**b**) boundary conditions; (**c**) von Mises stress amplitude distribution (unit: Pa); (**d**) max stress amplitude distribution in one cycle (unit: Pa); (**e**) max plastic strain amplitude distribution in one cycle.

In order to understand the above mention experimental phenomena and characteristics of LCF crack initiation positions and propagation paths of cast Mg-Al alloys (AM60B and AZ91) based on the fatigue damage or fatigue fracture theory, the finite element method (FEM) was carried out as a typical case in Figure 8 under one cyclic loading. The mechanical parameters such as the Young modulus and Poisson ratio and so forth, used in the finite element (FE) analysis are listed in Table 2, in which there is the roughly same relative ratio D-values (25.86% and 24.43%) of α-Mg and β-Mg_17_Al_12_ for the Young modulus [such as (*E*_β_−*E*_α_)/*E*_β_ = 25.86%] and the micro hardness. The difference in the mechanical properties both α-Mg and β-Mg_17_Al_12_ caused the mismatch of deformation and the fatigue cracks occurred easily at the interface [11,13,16,17,18,19]. In order to estimate quantitatively the mechanical responses, including stress and strain in the local region under remote loading, the FE modeling was carried out and the results are shown in Figure 8a,b. Figure 8c indicated that the maximum von Mises stress is mainly along tilted 45° and 90° directions development and the maximum stress value occurred always at the edge of hole. It means that the fatigue crack is the most likely to initiate at the edge of a hole, which is in good agreement with the experimental results as shown in Figure 3c, Figure 4c, Figure 5a,b, Figure 6a and Figure 7b–d. It has been indirectly proven that the LCF crack initiation behavior is dominated by the stress concentration at the edge of hole. However, the FE analyses as shown in Figure 8d–e indicated the maximum stress and deformation distribution characteristics exist slightly different around the hole under a cyclic stress, respectively. These differences can explain the LCF crack propagation paths of cast Mg-Al alloys by using the material fracture criterion or different strength theories. Compared with the maximum stress or plastic strain distribution characteristics of cast Mg-Al alloy with two through holes for 45° tilted to applied loading direction, the effective sharing part of the load is main the plastic strain as shown in Figure 8e. It hints that the fatigue multi-cracks occurred randomly in the region between the two small through holes and other fatigue cracks were amalgamated if the elongation of cast Mg-Al alloy was relative smaller. This is due to the fact that the effect of two small through holes on the fatigue crack propagation behavior cannot be ignored. The fatigue damage of cast Mg-Al alloy with a small elongation was dominated by the plastic strain which agrees with the experimental results.

## 5. Conclusions

The tests on the fatigue crack initiation and propagation of cast Mg-Al alloys with the two small holes were carried out by using in-situ SEM observation. The experimental results indicate that there are the critical geometric parameters of two small holes to influence on the fatigue behavior of cast Mg-Al alloys and agree with the finite element analysis. Main conclusions are obtained as follows:

1. For cast AZ91 alloy, the fatigue crack propagation behavior is more sensitive to changes in the distance between the two small holes than that of cast AM50 and AM60B alloys. This is because the plastic deformation capability of the former is much lower than that of the latter so that the LCF crack propagation resistance of the former is lower than that of the latter. The low cycle fatigue crack propagation behavior of the former depends strongly on the plastic strain or the maximum stress value but that of the latter depends mainly on the von Mises stress amplitude and its distribution.
2. The LCF crack initiation behavior of cast AM60B and AZ91 alloys with tilted 90° and the hole distances indicated that all fatigue crack initiation seats occurred at the edge of hole and LCF crack propagation behavior depends mainly on the distance of two holes, which the LCF crack propagates preferentially between two small through holes when the hole distance is less than λ = 2 mm (4D). Therefore, the correlative effect of the distance of about λ = 2 mm (4D) on the LCF crack propagation can be defined as the critical distance between two small holes when the diameter of hole is about 0.5 mm.
3. The LCF crack initiation behavior of cast Mg-Al alloys with tilted angle α = 45° and different hole distances is similar to that of the case with tilted angle α = 90° but the crack propagation behavior depends not only on the distance of two through holes but also on the plastic deformation capability of cast AM60B and AZ91 alloys as well as the applied stress ratio λ parameter (λ = σ_max_/σ_0.2_).
4. The LCF crack growth rates of the cast AZ91 and AM60B alloys are about 1.38 × 10^−8^ m/cycle and 0.83 × 10^−8^ m/cycle, respectively, under the same condition. Therefore, the fatigue life of the former is shorter (just under 2 times) than that of the latter. The results demonstrate a good correlation with S-N curves by using the longitudinal coordinates of the maximum stress amplitude or the λ parameter.

## Figures and Tables

**Table 1:** Chemical composition and mechanical properties of cast Mg-Al alloys (wt %) at room temperature.

Materials	Al	Mn	Si	Zn	Cu	Mg	σ_0.2_ (MPa)	σ_b_ (MPa)	Δ (%)
AM50	2.50	0.2	1.20	0.25	0.080	Bal.	140	200	15.0
AM60B	5.99	0.2	1.20	0.25	0.008	Bal.	150	160	10.0
AZ91	8.97	-	0.05	0.45	0.025	Bal.	160	240	3.5

**Table 2:** Mechanical properties of micro structure for AM60B alloy.

Materials	*E* (GPa)	υ	Micro Hardness (MPa)	Crystal Particle Size (μm)	FE Mesh
α-Mg	43	0.35	66.86	10	hexagon
β-Mg_17_Al_12_	58	0.30	88.34	15	hexagon
